# Perceptions of Health Misinformation on Social Media: Cross-Sectional Survey Study

**DOI:** 10.2196/51127

**Published:** 2024-04-30

**Authors:** Anna Gaysynsky, Nicole Senft Everson, Kathryn Heley, Wen-Ying Sylvia Chou

**Affiliations:** 1 Health Communication and Informatics Research Branch Behavioral Research Program National Cancer Institute Rockville, MD United States; 2 ICF Next ICF Rockville, MD United States

**Keywords:** social media, misinformation, health communication, health literacy, patient-provider communication

## Abstract

**Background:**

Health misinformation on social media can negatively affect knowledge, attitudes, and behaviors, undermining clinical care and public health efforts. Therefore, it is vital to better understand the public’s experience with health misinformation on social media.

**Objective:**

The goal of this analysis was to examine perceptions of the social media information environment and identify associations between health misinformation perceptions and health communication behaviors among US adults.

**Methods:**

Analyses used data from the 2022 Health Information National Trends Survey (N=6252). Weighted unadjusted proportions described respondents’ perceptions of the amount of false or misleading health information on social media (“perceived misinformation amount”) and how difficult it is to discern true from false information on social media (“perceived discernment difficulty”). Weighted multivariable logistic regressions examined (1) associations of sociodemographic characteristics and subjective literacy measures with misinformation perceptions and (2) relationships between misinformation perceptions and health communication behaviors (ie, sharing personal or general health information on social media and using social media information in health decisions or in discussions with health care providers).

**Results:**

Over one-third of social media users (35.61%) perceived high levels of health misinformation, and approximately two-thirds (66.56%) reported high perceived discernment difficulty. Odds of perceiving high amounts of misinformation were lower among non-Hispanic Black/African American (adjusted odds ratio [aOR] 0.407, 95% CI 0.282-0.587) and Hispanic (aOR 0.610, 95% CI 0.449-0.831) individuals compared to White individuals. Those with lower subjective health literacy were less likely to report high perceived misinformation amount (aOR 0.602, 95% CI 0.374-0.970), whereas those with lower subjective digital literacy were more likely to report high perceived misinformation amount (aOR 1.775, 95% CI 1.400-2.251). Compared to White individuals, Hispanic individuals had lower odds of reporting high discernment difficulty (aOR 0.620, 95% CI 0.462-0.831). Those with lower subjective digital literacy (aOR 1.873, 95% CI 1.478-2.374) or numeracy (aOR 1.465, 95% CI 1.047-2.049) were more likely to report high discernment difficulty. High perceived misinformation amount was associated with lower odds of sharing general health information on social media (aOR 0.742, 95% CI 0.568-0.968), using social media information to make health decisions (aOR 0.273, 95% CI 0.156-0.479), and using social media information in discussions with health care providers (aOR 0.460, 95% CI 0.323-0.655). High perceived discernment difficulty was associated with higher odds of using social media information in health decisions (aOR 1.724, 95% CI 1.208-2.460) and health care provider discussions (aOR 1.389, 95% CI 1.035-1.864).

**Conclusions:**

Perceptions of high health misinformation prevalence and discernment difficulty are widespread among social media users, and each has unique associations with sociodemographic characteristics, literacy, and health communication behaviors. These insights can help inform future health communication interventions.

## Introduction

### Background

The Pew Research Center estimates that approximately 72% of Americans use social media [1], and research suggests that social media is widely used for health-related purposes specifically [2]. Social media has become an important venue for the exchange of health-related information and advice [3]. In 2019, 41% of internet users in the United States reported watching health-related YouTube videos, and 17% reported sharing health information on social networking sites [4]. Social media can help people find and access more useful and personally relevant information, facilitate the exchange of social support, and aid with disease management efforts [3]. However, while social media can make health information more accessible, the use of social media for health information seeking can also create the risk of harm through exposure to misinformation.

Defined as “health-related information that is false, inaccurate, or misleading according to the best available evidence at the time,” health misinformation is increasingly recognized as a threat to public health [5,6] (note that this definition includes *disinformation*, or false information that is created and spread with the intent to deceive, as a subset of misinformation [7]). Although health misinformation is not a new phenomenon, social media facilitates the rapid spread of falsehoods [6], thereby exacerbating the potential negative impact of misinformation on both individual and population health. Certain features of social media platforms, such as incentives that reward the sharing of content that receives more engagement, can result in a focus on sharing emotionally charged or provocative content rather than accurate content [6,8]. Additionally, algorithms that suggest content to users are often based on past engagement behavior, which can reinforce echo chambers, whereby users who engage with misinformation increasingly encounter further misinformation [6,8].

Many studies have documented substantial health-related misinformation on social media across a range of topics (including tobacco products, drugs, and vaccines) [9], and research increasingly suggests that social media misinformation can have a negative impact on health-related attitudes, behaviors, and outcomes. For example, Pierri et al [10] found that the prevalence of COVID-19 vaccine misinformation on Twitter (now rebranded as X) was related to higher levels of vaccine hesitancy and lower vaccination uptake rates in the United States even after accounting for political and sociodemographic factors. Furthermore, their causality analysis suggested a directional relationship between social media misinformation and vaccine hesitancy, with a lag of approximately 2 to 6 days from misinformation being posted in a county to a corresponding increase in vaccine hesitancy in that county [10]. Further evidence of a causal relationship between exposure to misinformation and health-related attitudes and intentions is provided by a randomized controlled trial conducted in the United States and the United Kingdom, which showed that exposure to misinformation in the form of social media posts decreased the number of respondents who said that they would “definitely” take the COVID-19 vaccine by approximately 6 percentage points relative to the control group [11].

While there is a growing body of research examining the prevalence of misinformation on social media, as well as the association between social media misinformation and health-related outcomes, little work to date has focused on understanding how individuals perceive misinformation on social media or how these perceptions impact behavior. Surveys conducted over the past few years show that many US adults believe that much of the news they see on social media is false or inaccurate [12,13]. This is significant because studies have shown that misinformation perceptions impact communication behaviors. For example, a study conducted in Germany found higher self-perceived exposure to “fake news” to be associated with more frequent engagement in information verification behaviors on Facebook [14]. Meanwhile, a study conducted on Amazon Mechanical Turk found that perceiving fake news to have a greater influence on others than on oneself (ie, the third-person effect) was associated with lower intent to share news obtained from social media (either online or offline) [15].

Studies also suggest that perceptions of misinformation prevalence are associated with attitudes toward health issues—for example, one cross-sectional study found perceptions of high misinformation prevalence to be correlated with worry about COVID-19 [16]. Notably, the study found neither a significant association between actual misinformation prevalence (as measured using the “Infodemic Risk Index,” which produces frequency estimates of misinformation on Twitter by country) and worry about COVID-19 nor an interaction between actual misinformation prevalence and perceived prevalence in explaining pandemic worry [16]. This suggests critical psychological and cognitive effects of misinformation perceptions independent of actual misinformation prevalence (and separate from misinformation endorsement or belief) [16].

In addition to assessing perceptions of misinformation prevalence, assessing people’s confidence in their ability to detect misinformation is important because confidence can affect the way people make subsequent judgments [17]. For example, confidence can determine whether an individual acts on their initial judgment or seeks out additional information [18]. Confidence levels also affect a person’s willingness and ability to defend their assessments such that individuals who are able to discern true from false information—and are confident about their judgments—are more resistant to misinformation [18]. A person’s perceptions about their ability to accurately detect misinformation can also influence their perceptions about their capacity to manage health issues or make health decisions. For example, Park et al [19] found that people who had higher confidence in their ability to distinguish between true and false COVID-19 information also had higher COVID-19 risk readiness perceptions (ie, felt that they had a “handle on the issues and developments surrounding the coronavirus outbreak”). Unfortunately, confidence can also be easily undermined, particularly when an individual is unsure about the validity of the material they are considering or lacks the necessary skills or literacy competencies to feel secure in their assessment [17].

Beyond obtaining a better understanding of misinformation perceptions and how they impact cognitive and behavioral processes and outcomes, it is also important to assess whether these perceptions vary by sociodemographic or other characteristics to identify groups that may be more vulnerable to misinformation and in need of more targeted efforts. For example, the trial conducted by Loomba et al [11] showed that some groups were differentially affected by exposure to misinformation—in the United States, female individuals were found to be less resistant to misinformation than male individuals, whereas those with lower incomes were found to be more resistant. Additionally, a survey conducted by the Pew Research Center in 2016 found that White individuals were more likely than Black and Hispanic individuals to say that they often saw fake political news online, and those with annual incomes of at least US $75,000 were more likely to report seeing fake news compared to those who made less than US $75,000 per year [20]. Findings regarding demographic differences in perceived ability to discern true from false information are more mixed. The Pew survey found that confidence in detecting fake political news did not differ significantly by sociodemographic characteristics (such as age, gender, income, or race) [20], whereas the study conducted by Park et al [19] found education and income to be significant predictors of confidence in distinguishing true from false information about COVID-19. Furthermore, a large survey experiment conducted by Sirlin et al [21] found digital literacy (as measured by familiarity with internet-related terms and attitudes toward technology as well as understanding of social media algorithms) to be an important predictor of the ability to discern truths from falsehoods when judging headline accuracy for both political and COVID-19 articles. Taken together, these findings demonstrate the importance of assessing how perceptions of health misinformation and misinformation discernment vary by sociodemographic characteristics and literacy. Although research regarding vulnerability to misinformation remains mixed (eg, with regard to age, sex, and income) [22], the potential impact of social media misinformation on health disparities is an important issue that requires ongoing attention.

### Study Aims

Because misinformation perceptions can affect attitudes and behaviors, a better understanding of the public’s perceptions of health misinformation on social media and their ability to detect it, as well as possible subgroup differences in such perceptions, is needed. Toward that end, this study analyzed data from the National Cancer Institute’s 2022 Health Information National Trends Survey (HINTS 6) to (1) assess the prevalence of 2 distinct misinformation-related perceptions—perceived amount of health misinformation on social media and perceived ability to distinguish true from false health information on social media—(2) identify sociodemographic factors associated with these health misinformation perceptions; and (3) explore associations between these misinformation perceptions and health communication behaviors, including information sharing, health decision-making, and communicating with health care providers.

## Methods

### Data and Sample Selection

HINTS is a nationally representative, cross-sectional, self-administered survey of civilian, noninstitutionalized US adults aged ≥18 years. Data for HINTS 6 (N=6252) were collected between March 7, 2022, and November 8, 2022, using questionnaires administered via mailed paper or web-based surveys. The overall response rate for HINTS 6 was 28.1%. Respondents who reported that they did not use social media (1211/6252, 19.37%) were excluded from the analyses, resulting in a starting analytic sample of 5041. Details regarding the design of HINTS 6, including methodology, sampling, and weighting procedures, have been published elsewhere [23].

### Ethical Considerations

HINTS 6 received approval from the Westat Institutional Review Board on May 10, 2021 (6632.03.51), and was designated as non–human subjects research by the National Institutes of Health Office of Human Subjects Research on August 16, 2021 (000626). Respondents’ return of the completed survey indicated consent to participate.

### Measures

#### Social Media Health Misinformation Perceptions

A total of 2 social media misinformation–related perceptions were measured. Perceived amount of misinformation on social media (“perceived misinformation amount”) was assessed with the following item: “How much of the health information that you see on social media do you think is false or misleading?” Response options were *none*, *a little*, *some*, *a lot*, and *I do not use social media* (as noted previously, those who selected “I do not use social media” in response to this item were excluded from the analyses).

Perceived difficulty distinguishing true from false information on social media (“perceived discernment difficulty”) was measured by assessing agreement with the following statement—“I find it hard to tell whether health information on social media is true or false”—among respondents who reported social media use. Response options were *strongly agree*, *somewhat agree*, *somewhat disagree*, and *strongly disagree*.

#### Health Communication Behaviors Related to Social Media Use

Information sharing on social media was assessed using two items that asked how often in the previous 12 months respondents (1) “share[d] personal health information on social media” and (2) “share[d] general health-related information on social media (for example, a news article).” Response options were *almost every day*, *at least once a week*, *a few times a month*, *less than once a month*, and *never*.

Respondents’ use of information encountered on social media was assessed through reported agreement with 2 items: “I use information from social media to make decisions about my health” and “I use information from social media in discussions with my healthcare provider.” Response options were *strongly agree*, *somewhat agree*, *somewhat disagree*, and *strongly disagree*.

#### Sociodemographic Characteristics

Sociodemographic variables included (1) educational level (categorized as high school degree or lower, some college or vocational training, and college graduate or higher), (2) sex (male or female), (3) age (18-24 years, 25-34 years, 35-44 years, 45-54 years, 55-64 years, and ≥65 years), (4) race or ethnicity (non-Hispanic White; non-Hispanic Black/African American; Hispanic; and non-Hispanic other, which included non-Hispanic American Indian or Alaska Native, non-Hispanic Asian, non-Hispanic Native Hawaiian or other Pacific Islander, and non-Hispanic multiple races), (5) annual household income (<US $20,000, US $20,000-<$35,000, US $35,000-<$50,000, US $50,000-<$75,000, US $75,000-<$100,000, and ≥US $100,000), and (6) geographic residence (urban or rural based on the 2013 Rural-Urban Continuum Codes).

#### Literacy Measures

Subjective health literacy was assessed using the following item: “How confident are you filling out medical forms by yourself?” Response options were *very* [confident], *somewhat* [confident], *a little* [confident], and *not at all* [confident]. This measure is one of the brief screening questions identified by Chew et al [24] for detecting inadequate or marginal health literacy among adults. Subjective digital literacy was assessed using the following item: “How confident are you that you can find helpful health resources on the Internet?” Response options were *completely confident*, *very confident*, *somewhat confident*, *a little confident*, and *not at all confident*. This measure was adapted from the eHealth Literacy Scale [25]. Subjective numeracy was assessed using the following item: “In general, how easy or hard do you find it to understand medical statistics?” Response options were *very easy*, *easy*, *hard*, and *very hard*. This item, which is part of the STAT-Confidence scale developed by Woloshin et al [26], has been shown to be a strong predictor of scores on the Newest Vital Sign measure (an objective measure of health literacy and numeracy) [27].

### Statistical Analysis

To account for the complex sampling design of HINTS, analyses were conducted in SAS (version 9.4; SAS Institute) using final sample weights to obtain population-level point estimates and a set of 50 replicate weights to compute accurate variance estimates [23]. Frequencies and survey-weighted unadjusted proportions were used to describe the distributions of perceived misinformation amount and perceived discernment difficulty.

In total, 2 weighted multivariable logistic regression models examined associations of sociodemographic characteristics and literacy measures with perceived misinformation amount and perceived discernment difficulty. For these analyses, perceived misinformation amount was dichotomized to reflect high perceived misinformation amount (*a lot*) versus low perceived misinformation amount (*none*, *a little*, or *some*) to facilitate comparison between those who perceived misinformation to be a significant problem in the information environment and those who did not. Furthermore, only a relatively small proportion of respondents felt that “none” or only “a little” of the information they saw on social media was false or misleading, whereas over a third of the sample reported that “a lot” of the information they saw was false or misleading. Perceived discernment difficulty was dichotomized as high (*strongly agree* or *somewhat agree*) versus low (*somewhat disagree* or *strongly disagree*). Additionally, subjective health literacy was dichotomized as high (*very* [confident] or *somewhat* [confident]) versus low (*a little* [confident] or *not at all* [confident]), digital literacy was dichotomized as high (*completely confident* or *very confident*) versus low (*somewhat confident*, *a little confident*, or *not at all confident*), and numeracy was dichotomized as high (*very easy* or *easy*) versus low (*hard* or *very hard*).

A total of 4 additional weighted multivariable logistic regression models tested associations of high versus low perceived misinformation amount and high versus low perceived discernment difficulty with communication behaviors related to social media use (ie, sharing personal health information on social media, sharing general health information on social media, using information from social media to make health decisions, and using information from social media in discussions with health care providers) adjusted for sociodemographic characteristics and dichotomized literacy measures. The 2 information-sharing behavior measures were dichotomized as ever shared (*almost every day*, *at least once a week*, *a few times a month*, or *less than once a month*) versus never shared, whereas the 2 social media information use items were dichotomized as agreement (*strongly agree* or *somewhat agree*) versus disagreement (*somewhat disagree* or *strongly disagree*). Sensitivity analyses tested the interaction of perceived misinformation amount and discernment difficulty in predicting these communication outcomes, but this interaction was not statistically significant in any of the models.

Adjusted analyses used complete case analysis, with valid analytic samples reported in tables corresponding to each analysis. Descriptive information on missing data for each variable is publicly available on the HINTS website [28]. Tests of significance were conducted at the *P*<.05 level.

## Results

### Prevalence of Social Media Health Misinformation Perceptions

**Figure 1:**
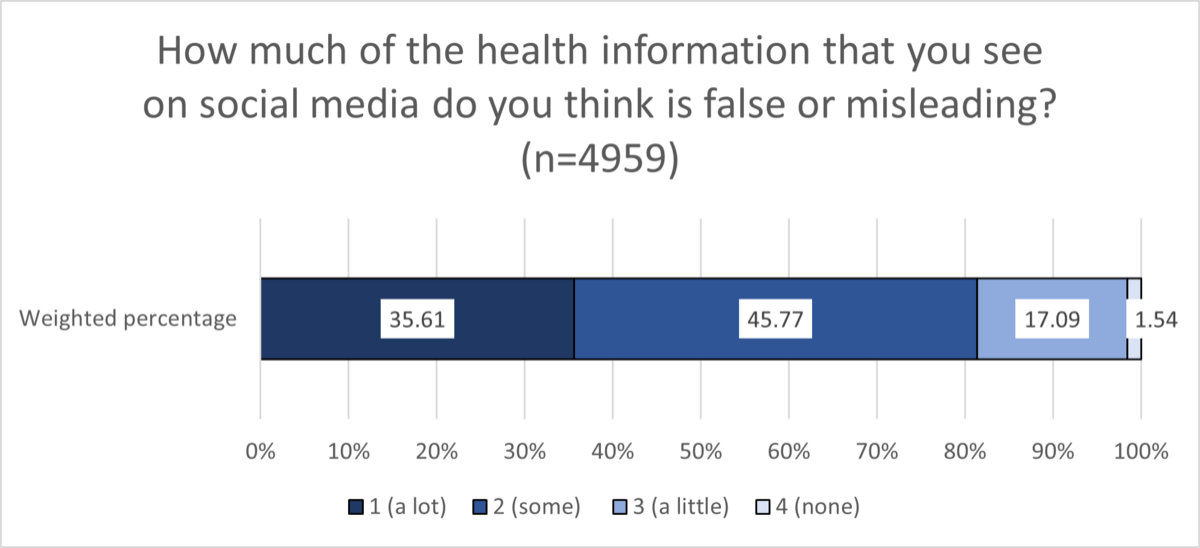
Weighted proportions of perceived health misinformation amount among American social media users.

**Figure 2:**
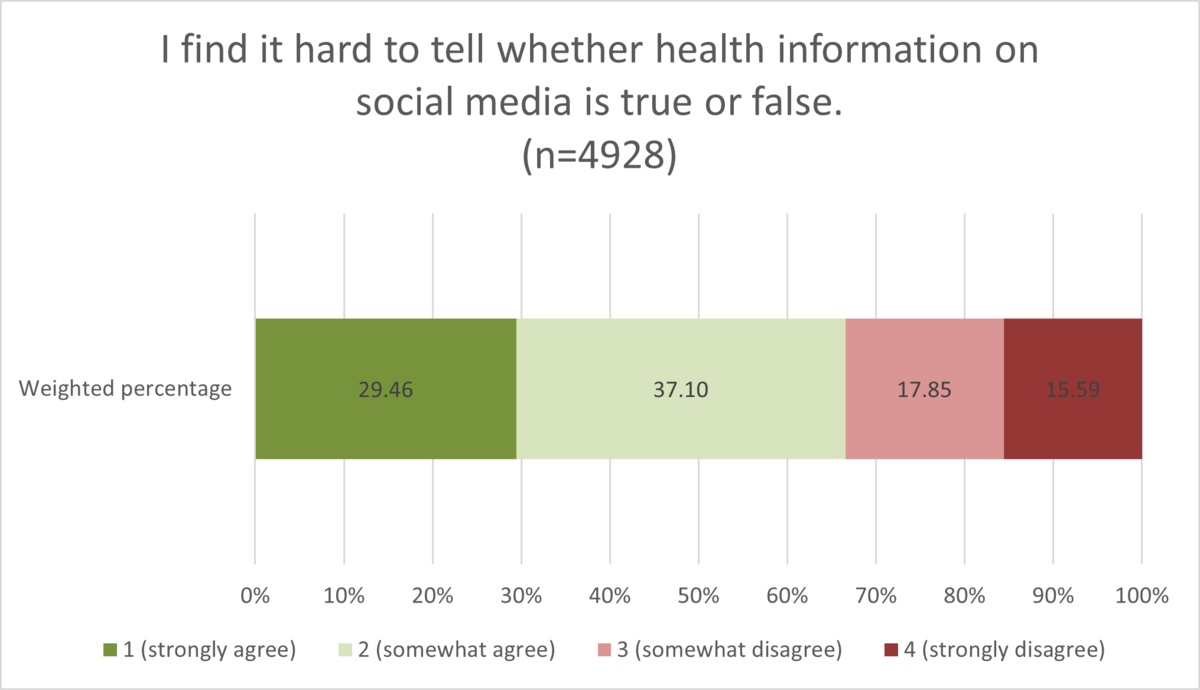
Weighted proportions of perceived discernment difficulty among American social media users.

As shown in Figure 1, over one-third of American social media users (35.61%) perceived “a lot” of misinformation on social media (ie, expressed high perceived misinformation amount), whereas only a very small percentage (1.54%) perceived that “none” of the health information they see is false or misleading. Figure 2 shows that approximately two-thirds of American social media users agreed that they find it hard to tell whether health information on social media is true or false (ie, endorsed high discernment difficulty).

### Predictors of Social Media Health Misinformation Perceptions

#### Perceived Misinformation Amount

As shown in Table 1, individuals who were non-Hispanic Black/African American (compared to non-Hispanic White individuals; adjusted odds ratio [aOR] 0.407, 95% CI 0.282-0.587) or Hispanic (compared non-Hispanic White individuals; aOR 0.610, 95% CI 0.449-0.831) or who had lower subjective health literacy (vs those with higher health literacy; aOR 0.602, 95% CI 0.374-0.970) were less likely to report high perceived misinformation amount. Comparatively, respondents with lower subjective digital literacy were more likely to report high misinformation amount (vs those with higher digital literacy; aOR 1.775, 95% CI 1.400-2.251). Age, sex, educational level, income, geographic residence, and numeracy were not statistically significantly related to perceived amount of misinformation.

**Table 1:** Predictors of social media health misinformation perceptions.

Independent variable		Perceived misinformation amount^a^ (n=4218), adjusted odds ratio (95% CI)	Perceived discernment difficulty^b^ (n=4205), adjusted odds ratio (95% CI)
**Age (y; reference: 18-24)**			
	25-34	0.745 (0.403-1.376)	*0.485* (0.289-0.816)^c^
	35-44	0.859 (0.495-1.491)	*0.487* (0.311-0.763)
	45-54	0.772 (0.410-1.454)	0.624 (0.384-1.014)
	55-64	0.790 (0.425-1.468)	*0.605* (0.369-0.990)
	≥65	0.688 (0.384-1.232)	0.841 (0.525-1.346)
**Sex (reference: male)**			
	Female	1.037 (0.791-1.358)	1.077 (0.871-1.331)
**Educational level (reference: high school or lower)**			
	Some college or vocational training	1.206 (0.867-1.677)	1.210 (0.882-1.660)
	College graduate or higher	1.144 (0.809-1.618)	0.871 (0.632-1.200)
**Race or ethnicity (reference: non-Hispanic White)**			
	Hispanic	*0.610* (0.449-0.831)	*0.620* (0.462-0.831)
	Non-Hispanic Black/African American	*0.407* (0.282-0.587)	0.830 (0.596-1.156)
	Non-Hispanic other	0.977 (0.662-1.442)	1.126 (0.788-1.607)
**Annual household income (reference: <US $20,000)**			
	US $20,000-$34,999	1.283 (0.747-2.202)	1.016 (0.640-1.614)
	US $35,000-$49,999	1.039 (0.613-1.760)	1.061 (0.678-1.659)
	US $50,000-$74,999	1.619 (0.968-2.709)	1.058 (0.740-1.512)
	US $75,000-$99,999	1.693 (0.996-2.880)	1.459 (0.932-2.283)
	≥US $100,000	1.469 (0.910-2.369)	1.245 (0.870-1.780)
**Geographic residence (reference: urban)**			
	Rural	1.012 (0.770-1.331)	1.109 (0.769-1.600)
**Health literacy (reference: high health literacy)**			
	Low health literacy	*0.602* (0.374-0.970)	1.230 (0.829-1.824)
**Digital literacy (reference: high digital literacy)**			
	Low digital literacy	*1.775* (1.400-2.251)	*1.873* (1.478-2.374)
**Numeracy (reference: high numeracy)**			
	Low numeracy	1.030 (0.771-1.376)	*1.465* (1.047-2.049)

^a^The probability modeled was odds of high perceived misinformation amount (*a lot*) in reference to low perceived misinformation amount (*none*, *a little*, or *some*).

^b^The probability modeled was odds of reporting high perceived discernment difficulty (*strongly agree* or *somewhat agree*) in reference to low perceived discernment difficulty (*somewhat disagree* or *strongly disagree*).

^c^Italicized values are statistically significant (*P*<.05).

#### Perceived Discernment Difficulty

As shown in Table 1, there were differences in perceived discernment difficulty by age—adults aged 25 to 34 years, 35 to 44 years, and 55 to 64 years were less likely to report high discernment difficulty compared to those aged 18 to 24 years, whereas adults aged 45 to 54 years and those aged ≥65 years did not differ significantly from the youngest age group. Hispanic individuals (vs non-Hispanic White individuals; aOR 0.620, 95% CI 0.462-0.831) were less likely to report high discernment difficulty. Those with lower (vs higher) subjective digital literacy (aOR 1.873, 95% CI 1.478-2.374) or lower (vs higher) subjective numeracy (aOR 1.465, 95% CI 1.047-2.049) were more likely to report high discernment difficulty. The associations between perceived discernment difficulty and sex, educational level, income, geographic residence, and health literacy were not statistically significant.

### Associations Between Social Media Health Misinformation Perceptions and Communication Behaviors

After adjusting for sociodemographic characteristics and literacy measures, individuals who perceived high (vs low) levels of social media misinformation were less likely to report sharing general health information on social media (aOR 0.742, 95% CI 0.568-0.968), using social media information to make health decisions (aOR 0.273, 95% CI 0.156-0.479), and using social media information in discussions with health care providers (aOR 0.460, 95% CI 0.323-0.655). Perceived misinformation amount was not significantly associated with sharing personal health information on social media (Table 2).

Individuals with high (vs low) perceived discernment difficulty were more likely to report using information from social media to make health decisions (aOR 1.724, 95% CI 1.208-2.460) and in discussions with health care providers (aOR 1.389, 95% CI 1.035-1.864). Perceived discernment difficulty was not significantly associated with sharing personal or general health information on social media.

**Table 2:** Adjusted odds ratio (aOR) and 95% CI of health information sharing and social media information use by social media health misinformation perceptions^a^.

Social media misinformation perception	Sharing personal health information^b^ (n=4136), aOR (95% CI)	Sharing general health information^c^ (n=4159), aOR (95% CI)	Using social media information to make health decisions^d^ (n=4177), aOR (95% CI)	Using social media information in discussions with health care providers^e^ (n=4174), aOR (95% CI)
High perceived misinformation amount^f^	0.803 (0.591-1.092)	*0.742* (0.568-0.968)^g^	*0.273* (0.156-0.479)	*0.460* (0.323-0.655)
High perceived discernment difficulty^h^	1.163 (0.862-1.570)	1.100 (0.878-1.379)	*1.724* (1.208-2.460)	*1.389* (1.035-1.864)

^a^Analyses were adjusted for age, sex, educational level, race or ethnicity, income, geographic residence, health literacy, digital literacy, and numeracy.

^b^The probability modeled was odds of having ever shared personal information on social media (*shared almost every day, at least once a week, a few times a month*, or *less than once a month in the past 12 months*) in reference to having never shared.

^c^The probability modeled was odds of having ever shared general information on social media (*shared almost every day, at least once a week, a few times a month*, or *less than once a month in the past 12 months*) in reference to having never shared.

^d^The probability modeled was odds of using social media information for making health decisions (*strongly agree* or *somewhat agree*) in reference to not using social media information for making health decisions (*strongly disagree* or *somewhat disagree*).

^e^The probability modeled was odds of using social media information in discussions with health care providers (*strongly agree* or *somewhat agree*) in reference to not using social media information in discussions with health care providers (*strongly disagree* or *somewhat disagree*).

^f^High perceived misinformation amount=thinking that *a lot* of the health information on social media is false or misleading; low perceived misinformation amount=thinking that *none*, *a little*, or *some* of the health information on social media is false or misleading.

^g^Italicized values are statistically significant (*P*<.05).

^h^High perceived discernment difficulty=strongly or somewhat agreeing that it is hard to tell whether health information on social media is true or false; low perceived discernment difficulty=strongly or somewhat disagreeing that it is hard to tell whether health information on social media is true or false.

## Discussion

### Principal Findings

This study examined 2 misinformation-related perceptions among social media users (perception of the amount of health misinformation on social media and perceived ability to distinguish true from false health information on social media) to better understand the prevalence of these perceptions, subgroup differences in these perceptions, and how these perceptions are related to health communication behaviors. The study found that over one-third of social media users perceived their information environment to contain “a lot” of misleading or false content, and two-thirds expressed difficulty discerning true from false information on social media, with significant variation in these perceptions by sociodemographic characteristics and self-reported literacy skills. The analysis also showed that perceiving a high amount of misinformation on social media was related to lower information sharing on social media and lower use of social media information in discussions with providers and in health decisions, whereas difficulty distinguishing true from false information was associated with higher use of social media information in discussions with providers and health decisions. These results suggest that understanding misinformation perceptions could help inform health communication interventions and efforts to mitigate the impact of web-based misinformation, and that different approaches may be needed in response to each of these misinformation perceptions.

A substantial proportion of American social media users reported that “a lot” of the health information they see on social media is false or misleading, and this perception varied by race and ethnicity, as well as subjective measures of literacy. Non-Hispanic Black/African American and Hispanic individuals were less likely to say that “a lot” of the health information they see on social media is false or misleading. Because this analysis relied on self-report measures, it is not possible to ascertain whether minority groups are actually less exposed to social media misinformation (eg, due to the nature of their web-based networks) or if they are less aware that the information they are seeing is, in fact, false. However, the reasons behind these differences in misinformation perceptions and the potential for these differences to exacerbate health disparities deserve further attention given that Black and Hispanic individuals use social media at higher rates than White individuals [1] and substantial proportions of individuals in these groups report regularly obtaining their news from social media platforms [29]. Additional research that attempts to triangulate user perceptions with the social media content they encounter [30] could help shed light on the unique impact of objective and subjective social media experiences.

The analysis also found that individuals with lower digital literacy were more likely to report that “a lot” of the health information they see on social media is false or misleading, whereas those with lower health literacy were less likely to do so. This may be because individuals who self-report low confidence in their ability to find helpful resources on the web are more aware of content quality issues on the internet (including on social media), whereas reporting low subjective health literacy (eg, expressing difficulty filling out medical forms) may not be similarly related to concerns about the online information environment. In fact, a small study conducted in Europe found that participants with low health literacy (as measured using the Newest Vital Sign) had higher scores on the eHealth Literacy Scale, suggesting that they perceived themselves to have higher digital literacy than those in the high health literacy group [31]. The authors hypothesized that this finding might reflect differences in awareness of the issue of web-based health information quality between those with high versus low health literacy as well as differences in knowledge and use of established information evaluation criteria [31].

This study also revealed that approximately two-thirds of American social media users find it hard to tell whether health information on social media is true or false. High levels of discernment difficulty among the public are concerning. Low confidence in one’s ability to distinguish true from false information could result in lower motivation to seek additional information [19], apathy, and confusion, which could lead to negative health outcomes not just because people might act on misinformation but also because they may fail to act on accurate information or adhere to public health recommendations. Research suggests that self-efficacy (ie, judgments regarding how well one can execute a course of action required to deal with a prospective situation) plays an important role in how people select and evaluate information in web-based environments [32]. Individuals with higher self-efficacy may be better able to make accurate credibility assessments because they are more motivated to engage in deep cognitive processing and critical thinking [32], whereas those with lower self-efficacy may avoid engaging in extensive evaluations of information credibility, especially in contexts characterized by uncertainty and ambiguity, as they may not feel that they have a high likelihood of achieving desirable outcomes and, therefore, may experience negative affect (eg, anxiety, frustration, and confusion) in response to these situations [32]. However, although some research suggests that confidence in one’s ability to spot misinformation is associated with better performance in accurately distinguishing false from accurate news [32], the evidence is somewhat limited, and further research combining both subjective perceptions of ability and objective measures of ability is needed in order to investigate the impact of confidence on the way in which individuals navigate health information on social media.

Beyond generally high rates of discernment difficulty, this analysis also identified differences in perceived discernment ability in certain demographic subgroups. Specifically, adults aged 25 to 34 years, 35 to 44 years, and 55 to 64 years were less likely than those in the youngest age group to report discernment difficulty, and Hispanic individuals reported less discernment difficulty compared to non-Hispanic White individuals. Higher confidence in discernment ability among these groups could be justified (eg, slightly older adults may be just as technologically savvy as young adults but also have more experience and therefore may be better equipped to make accurate credibility assessments); however, it is also possible that discernment confidence in these groups is misplaced, which would be a cause for concern as it might mean that individuals in these groups are less likely to verify information that might be false (eg, through additional research or by speaking to a health care provider), potentially putting them at greater risk of acting on false information. Additional research is needed to better understand why these groups express higher levels of confidence in their discernment ability.

In contrast, those with lower digital literacy and those with lower numeracy were more likely to report high discernment difficulty. This is perhaps not surprising as research has shown lack of digital literacy to be associated with lower objective ability to successfully judge the accuracy of news stories [21], suggesting that individuals with lower digital literacy may be aware of their limitations in this area. Therefore, digital literacy skills as well as health information evaluation abilities may be important targets for interventions seeking to increase resiliency against misinformation—particularly among more susceptible groups.

The results of this analysis also indicated an association between health misinformation perceptions and distinct behavioral patterns. For example, individuals who perceived high levels of misinformation were less likely to share general health information on social media (perhaps because they are more aware of the problem and are more hesitant to share information that could be false), whereas self-reported discernment difficulty was not similarly associated with sharing behaviors on social media. This finding is in line with the results of previous research showing that confidence in one’s ability to identify factually incorrect information is not significantly associated with likelihood of sharing misinformation [32,33]. Some studies suggest that accuracy may not be the most important factor that people consider when making sharing decisions [34,35], which may help explain why uncertainty about the veracity of information does not significantly influence sharing behavior. Research has shown that interventions that prime individuals to consider accuracy when making sharing decisions on social media could be a promising way to mitigate the spread of misinformation [35], and the results of this analysis suggest that this strategy deserves further attention.

Additionally, the results of this study showed that individuals who perceived high levels of misinformation were less likely to use social media information in making health decisions or in discussions with health care providers. It is possible that, because these individuals perceive high amounts of health misinformation on social media, they are skeptical of the information they encounter on these platforms and, therefore, do not rely on it to inform either their conversations with health care providers or their health decision-making. In contrast, individuals who reported difficulty distinguishing between true and false information on social media were more likely to use information from these platforms in making health decisions and in discussions with health care providers, perhaps because they seek assistance from their health care providers in assessing the credibility of the information. These findings were somewhat counterintuitive, and future research exploring *how* and *why* individuals who report high discernment difficulty use the information they encounter on social media in health-related decisions and discussions could help provide important insights that are beyond the scope of this analysis. For example, exploring whether these individuals are asking for clarification about social media information in discussions with providers versus seeking a “second opinion” on social media after speaking to their clinicians would provide important context to these findings and could help inform how providers can best communicate with patients about information obtained from social media.

### Significance

This study offers a unique contribution to our understanding of social media misinformation by focusing on perceptions of the issue rather than objective assessments of misinformation prevalence, exposure, endorsement, or discernment. Assessing perceptions is important because perception of widespread misinformation on social media, as well as perceptions of personal ability to navigate misinformation in web-based spaces, can affect attitudes and behaviors—over and above the impact of actual exposure or ability [16]. In fact, individuals who report high perceived misinformation are likely less susceptible to the direct effects of misinformation (as individuals who characterize a claim as “misinformation” are unlikely to accept it or act on it); however, as demonstrated in this study as well as in previous work, misinformation perceptions can still shape their responses and behaviors [16].

Notably, there are limitations to using self-reported measures of perception—for example, it is impossible to know whether people’s perceptions are an accurate reflection of “the ground truth” (ie, whether a lot of the social media information they are exposed to really is or is not false and whether they are really capable of discerning the veracity of social media information) [20]. However, there is still value in assessing these perceptions to obtain a high-level understanding of the public’s views on the scope of the problem and the extent to which it affects them as well as their judgment of their own capacity to cope with the problem. In the context of political misinformation, individuals who perceived a lot of exposure to misinformation were more likely to believe that misinformation is a serious problem that creates a lot of confusion about the basic facts of current issues and events and were also more confident in their ability to identify misinformation [20]. Perceptions of the information environment can also impact attitudes and behaviors in ways that are important to health [19]—for example, people may feel overwhelmed and discouraged from seeking additional information about a health topic or develop inaccurate risk perceptions. Additionally, the differences in misinformation perceptions by demographics and literacy levels identified in this study are concerning as they threaten to increase disparities among vulnerable populations. However, while perceptions are important in and of themselves, future research could benefit from including both subjective and objective measures of the information environment to better understand the unique contribution of each construct and provide a more comprehensive understanding of how people respond to social media information.

Nonetheless, the results of this study suggest several practical measures that could help mitigate the impact of misinformation on social media. First, they point to specific populations that may benefit from targeted interventions. For example, those who perceived “a lot” of misinformation on social media were less likely to use this information in health decision-making, suggesting that interventions that raise awareness of information quality issues on social media could limit the extent to which individuals rely on questionable information from social media to make health decisions. These efforts may be especially impactful among groups who are less likely to report perceiving high amounts of misinformation on social media (eg, Black/African American and Hispanic individuals and individuals with lower health literacy). Additionally, the finding that those who express high discernment difficulty still use information from social media to make health decisions suggests that these individuals should be targeted for training interventions that can increase their ability to discern misinformation to (1) ensure that they are relying on accurate information to make these decisions and (2) increase their confidence in their ability to navigate the social media information environment. For example, instructional programs that train people to recognize misinformation techniques have been shown to increase their awareness of these tactics as well as confidence in their ability to successfully deal with misinformation [36]. Furthermore, increasing confidence in discernment ability may itself be a viable target for encouraging careful evaluation of information and increasing resilience to misinformation. For example, Ferrucci and Hopp [37] found that a short intervention providing positive verbal persuasion regarding participants’ ability to identify false information on social media increased fake news self-efficacy and that higher self-efficacy beliefs were in turn associated with ability to correctly classify both credible and “fake” news headlines in an information accuracy assessment task.

Second, the finding that those who express high discernment difficulty are more likely to have discussions with health care providers regarding social media health information suggests a need for training aimed at providers to support them in effectively helping patients navigate the information they encounter on the web (eg, teaching providers how to invite these conversations, address misinformation with empathy, and empower patients by recommending accurate sources of information) [38,39]. Research suggests that providers rarely initiate conversations about web-based health information seeking with patients [40,41], but the results of this study indicate that asking about patients’ perceptions and use of social media health information could be helpful to incorporate into patient-provider conversations.

Although this study looks at individual-level perceptions and has implications for individual-level interventions (eg, increasing digital literacy), the onus should not be solely on individuals (or providers) to address the problem of social media misinformation. Social media platforms could take steps to decrease the amount of misinformation that users are exposed to in the first place and make it easier for them to discern true from false information (eg, through the use of fact-checking labels and account verification). However, in the absence of these types of more systematic changes in the social media environment, individuals will likely be left to navigate the increasingly confusing information landscape on their own and will need to be supported in their efforts, for example, through campaigns to raise awareness of the issue (particularly among vulnerable populations), training on information evaluation strategies and common misinformation techniques, and encouragement to discuss social media health information with providers and others with relevant expertise. These interventions can be deployed in both web-based and offline contexts (eg, through video advertisements on social media platforms [42] or through educational services delivered in health care settings [43]).

### Limitations

This study has several limitations. First, the cross-sectional nature of HINTS data precludes causal inferences from being drawn about observed relationships between variables. Second, the misinformation measures included in this analysis are subjective perception items. As such, there is no way to determine the objective truth about a respondent’s actual misinformation exposure or their ability to differentiate true from false information. However, even if they do not reflect objective reality, perceptions are valuable to assess because they enable a better understanding of the public’s views on the scope of the misinformation problem and their capacity to cope with it and can help shed light on the way in which perceptions of the information environment shape health-related attitudes and behaviors. Third, the lack of information on certain aspects of respondents’ social media experiences and behaviors (eg, the specific platforms they use) is a limitation of this analysis—and reflects a disadvantage of using a national health communication survey that includes only a limited number of items regarding social media use due to space constraints. Finally, the response rate for HINTS 6 (28.1%) was relatively low, which may introduce bias into the data [44]. However, methodological research suggests that the impact of low response rates on data quality may be less significant than previously assumed [44]. Despite these limitations, this analysis provides an important contribution to the broader health misinformation literature as there has been limited research to date focusing on perceptions of misinformation, particularly outside the context of COVID-19.

### Conclusions

Many social media users in the United States perceive high levels of misinformation on social media and report difficulty discerning true from false information. This is concerning because perceptions of high misinformation prevalence could increase negative affect (eg, anxiety and worry) regarding health issues, whereas low discernment confidence could result in apathy, confusion, and lower motivation to seek additional information. The fact that health misinformation perceptions were found to vary across race, ethnicity, age, and literacy levels may suggest a need to raise awareness about misinformation and provide training for certain populations (eg, those with low health literacy) to ensure that they approach the information environment with sufficient skepticism and are better able to verify the health claims they see on social media. Finally, the associations between misinformation perceptions and social media–related communication behaviors found in this study can help inform future research as well as health communication interventions and misinformation mitigation efforts. For example, the finding that individuals who have low confidence in their discernment ability are more likely to use social media information to make health decisions and in discussions with health care providers suggests that they may benefit from providers assisting them in navigating and verifying web-based information.

Although a growing body of literature focusing on social media misinformation has emerged in recent years, to date, very little work has been done to look at subjective assessments of the problem of misinformation. This study provides initial insights into the prevalence, disparities, and potential impact of social media misinformation perceptions. However, more research is needed to understand how perceptions of misinformation affect the public’s health-related cognitions, attitudes, communication behaviors, and outcomes.

## Abbreviations

aOR: adjusted odds ratio
HINTS: Health Information National Trends Survey
